# Microarray and whole-exome sequencing analysis of familial Behçet’s disease patients

**DOI:** 10.1038/srep19456

**Published:** 2016-01-20

**Authors:** Daisuke Okuzaki, Kazuyuki Yoshizaki, Toshio Tanaka, Toru Hirano, Kohshiro Fukushima, Takanori Washio, Hiroshi Nojima

**Affiliations:** 1 DNA-chip Development Center for Infectious Diseases, Research Institute for Microbial Diseases, 3-1 Yamadaoka, Suita, Osaka 565-0871, Japan; 2 Department of Molecular Genetics, Research Institute for Microbial Diseases, Osaka University, 3-1 Yamadaoka, Suita, Osaka 565-0871, Japan; 3 Department of Organic Fine Chemicals, The Institute of Scientific and Industrial Research, Osaka University, 6-2-3 Furuedai, Suita, Osaka 565-0874, Japan; 4 Department of Clinical Application of Biologics, Osaka University, 2-2 Yamadaoka, Suita, Osaka 565-0871, Japan; 5 Department of Respiratory Medicine, Allergy and Rheumatic Diseases, Osaka University Graduate School of Medicine, Osaka University, 2-2 Yamadaoka, Suita, Osaka 565-0871, Japan; 6 Riken Genesis, Co. Ltd., Riken Yokohama Institute, 1-7-22 Suehiro-cho, Tsurumi-ku, Yokohama-shi, Kanagawa 230-0045, Japan

## Abstract

Behçet’s disease (BD), a chronic systemic inflammatory disorder, is characterized by recurrent oral and genital mucous ulcers, uveitis, and skin lesions. We performed DNA microarray analysis of peripheral blood mononuclear cell (PBMC) mRNA from 41 Japanese BD patients and revealed elevated levels of interleukin (IL) 23 receptor (*IL23R*) mRNA in many BD patients. DNA sequencing around a SNV (Rs12119179) tightly linked to BD revealed an elevated frequency of the C genotype, consistent with a previous report that *IL23R* is a susceptibility locus for BD. Notably, four of these BD patients are members of familial BD; a whole-exome sequencing (WES) of these BD patients identified 19 novel single-nucleotide variations (SNVs) specific to these patients. They include heterozygous SNVs in the genes encoding IL-1 receptor-associated kinase 4 (*IRAK4*), nucleotide-binding oligomerization domain (NOD)-like receptor family pyrin domain-containing 14 (*NRP14*) and melanoma antigen-encoding gene E2 (*MAGEE2*); *IRAK4* harbors a missense mutation, whereas *NRP14* and *MAGEE2* harbor nonsense mutations. These SNVs may serve as genetic markers that characterize BD.

Behçet’s disease (BD) is a chronic systemic inflammatory disorder characterized by four major manifestations: recurrent oral and genital mucous ulcers, uveitis, and skin lesions^1^,^2^. Because BD is more prevalent in certain geographical regions, with the highest incidence in countries along the ancient silk route spanning from Mediterranean countries to the Middle East and Japan, putative genetic variants and specific environmental factors are considered to be important for its etiology^2^. Across multiple ethnicities, many BD patients have the HLA-B*51 variant of the human leukocyte antigen (HLA) class I allele^3^. Because many healthy people also have this HLA-B51 variant, other genetic variations and/or environmental factors must be required to fully explain the etiology of BD.

Genome-wide association studies (GWAS) carried out in Turkey and Japan demonstrated that HLA-B*51, HLA-A*26, *IL10*, and *IL23R*–*IL12RB2* are susceptibility loci for BD^4^,^5^, and also revealed an interaction between HLA-B*51 and *ERAP1*^6^ HLA-Bw4-80I, present on HLA-B*51 and HLA-A*26, is an additional BD susceptibility marker^7^. GWAS in a Chinese cohort confirmed the association between *IL10* polymorphisms and BS^8^. In addition, single-nucleotide polymorphism (SNP) mapping of MHC with logistic regression revealed that the *HLA-B*/*MICA* region and the region between *HLA-F* and *HLA-A* are independently associated with BD^3^.

A GWAS in a Korean population identified a novel BD-associated locus encompassing the gene encoding GTPases of immunity-associated protein (*GIMAP*), which plays a role in peripheral T-cell function^9^. However, the *GIMAP* association did not replicate in European BD patients^10^. High copy-number variations (CNVs) of complement component C4A confer a risk for BD, and serum C4 protein level is significantly elevated in Chinese BD patients^11^. Non-synonymous variants identified by deep exonic resequencing confirmed the association of *IL23R* and *TLR4* with BD, suggesting the involvement of innate immune and bacterial sensing mechanisms in BD pathogenesis^12^.

In this study, we sought to identify genes whose mRNA levels were commonly up- or down-regulated in peripheral blood mononuclear cells (PBMCs) of BD patients, because such genes may be directly linked to the pathogenesis of BD. We identified several genes, including *IL23R*, that were commonly up-regulated in all BD patients examined. Moreover, we identified a rare case of familial BD over three generations, and performed genome-wide exome analysis to identify genes specific to these patients. Based on our results, we propose a novel model for the pathogenesis of BD.

## Results

### DNA microarray analysis of PBMCs from BD patients

**Figure 1:**
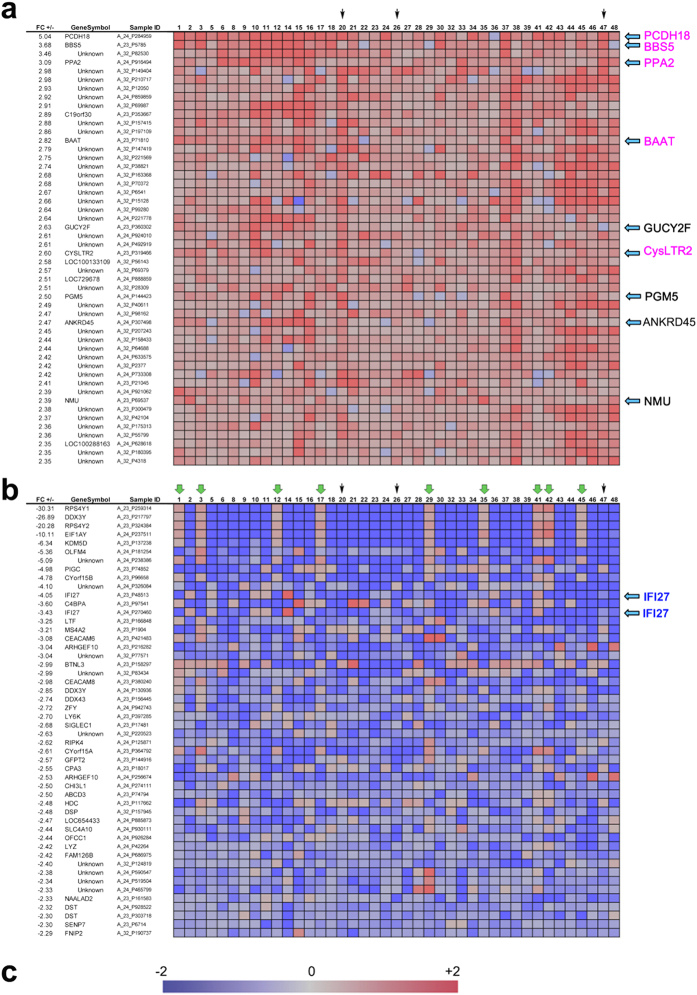
Expression profiles of genes whose mRNA levels were commonly up- or down-regulated in the PBMCs of 41 BD patients relative to those of healthy volunteers. Agilent’s whole human genome DNA microarray (Hu44K) was used for this analysis. (**a**) List of top 50 genes up-regulated in most BD patients is shown in decreasing order of fold-change values. (**b**) List of bottom 50 genes down-regulated in most BD patients is shown in increasing order of fold-change values. “Unknown” indicates uncharacterized genes. Vertical green arrows show that they are male patients. Agilent’s sample ID is presented for identification of probes used for the microarray analysis. Mosaic tile representation of each gene is also shown, with intensity gradients indicating the mean value of the expression level (log_2_ ratio): blue (down-regulation) and crimson (up-regulation) relative to the average value in healthy volunteers (gray). Names of notable genes are highlighted in larger font with blue arrows; genes also appearing in Figures S2 and/or S3 are shown in pink font. Vertical black arrows indicate familial BD patients (see Fig. 2a). (**c**) Bar represents the standard intensity gradient.

To determine whether gene expression profiles of many BD patients share common abnormalities relative to those of healthy volunteers (HVs), we performed genome-wide complementary DNA microarray analyses using an Agilent Hu44K array. RNA samples were isolated from PBMCs of 41 individual BD patients (32 females and nine males) and 17 HVs (Supplementary Fig. S1). Because we wanted to obtain putative gene markers that distinguish BD from healthy controls even during its inactive phase, BD patients were examined regardless of symptoms or disease activity/inactivity. When we arranged the top 50 up-regulated genes according to decreasing fold-change values (Fig. 1a) or the bottom 50 down-regulated genes according to increasing fold-change values (Fig. 1b), we found several genes were up-regulated (>3.0-fold, including *PCDH18*, *BBS5*, and PP2A), or down-regulated (<20-fold, including *RPS4Y1, DDX3Y* and *RPS4Y2*) in many BD patients.

However, several down-regulated genes tended to be up -regulated when only male patients were considered (green arrows in Fig. 1b), suggesting that some up- or down-regulated genes in the microarray data were gender-specific, which could have hampered the comprehensive analysis of the data. Moreover, the number of female patients (32) was much larger than that of male patients (nine). To prevent other important genes from being overlooked, we also arranged the data according to decreasing or increasing fold change values for male patients (Supplementary Fig. S2) and female patients (Supplementary Fig. S3). These analyses identified other markedly up-regulated genes, including *BAAT*, *CYSLTR1*, *IL23R, NMU* and *GUCYTF* (Supplementary Fig. S2a, S3a), or down-regulated genes, including *IFI27, OLFM4, NS4A2* and *PIGC* (Supplementary Fig. S2b, S3b). *IL23R*, in particular, is important because SNPs in the intergenic region between *IL23R* and *IL12RB2* is associated with BD^4^,^5^. The putative functions of these genes in the pathogenesis of BD are discussed in Supplementary Results. It is remarkable that many of the identified genes are unknown; this suggests that much remains to be done to determine the genetic basis of BD.

### DNA sequencing at the *IL23R–IL12RB2* locus for familial BD patients

Notably, *IL23R* mRNA was up-regulated in 39 of 41 BD patients (Fig. S2a). Previous GWAS identified associations of BD with SNPs in the intergenic region between *IL23R* and *IL12RB2*^4^,^5^. *IL23R* is also a susceptibility locus for a number of inflammatory and immune-linked diseases, including inflammatory bowel disease^13^, psoriasis^14^, psoriatic arthritis^15^, ankylosing spondylitis^16^, acute anterior uveitis^17^, Vogt-Koyanagi-Harada syndrome^18^, and idiopathic achalasia^19^. These findings suggest that up-regulation of IL23R is involved in the pathogenesis of BD.

**Figure 2:**
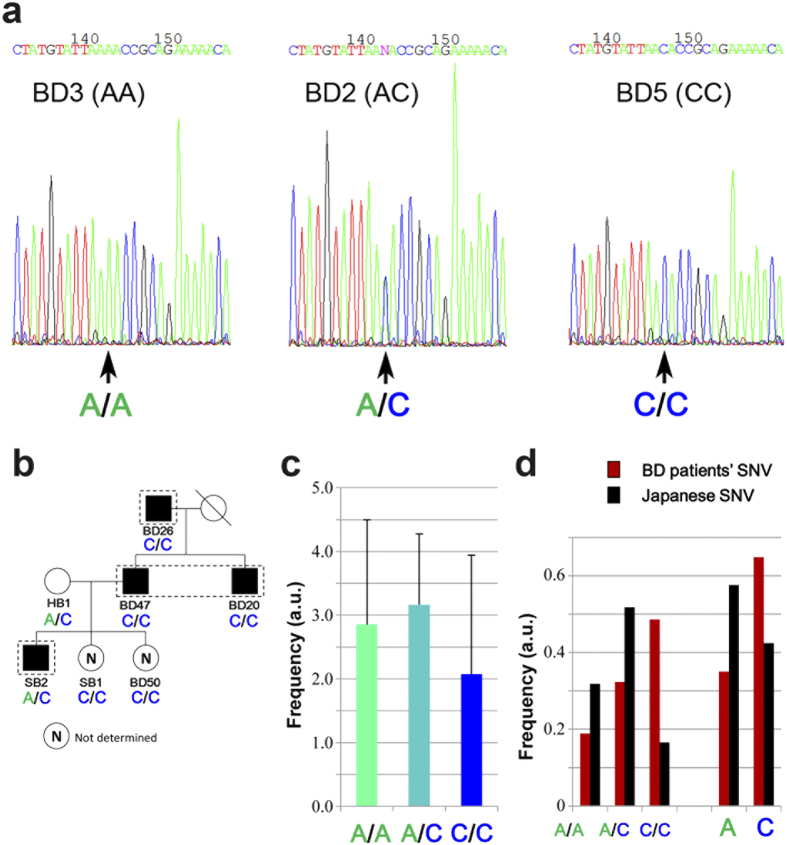
DNA sequences at the *IL23R*–*IL12RB2* locus of our BD patients. (**a**) DNA sequence ladder around the SNV (Rs12119179) for BD3, BD2, and BD5. Arrows indicate signal peaks for A/A, A/C, and C/C genotypes, respectively. (**b**) Genotypes of the Rs12119179 SNV for the BD family members. (**c**) Comparison of *IL23R* mRNA levels (see Fig. 1A) for BD patients with A/A, A/C, and C/C genotypes. (**d**) Frequencies of A/A, A/C, and C/C genotypes of SNV (Rs12119179) in our BD patients (red bars) or the healthy Japanese population (black bars). Frequencies of A and C populations calculated from A/A, A/C, and C/C genotypes are shown.

To determine whether up-regulation of *IL23R* mRNA levels was due to the SNV at a susceptibility locus for BD^4^,^5^, we determined the DNA sequence around the variant nucleotide (Rs12119179). BD patients had a variety of allele combinations (Supplementary Fig. S4a), including the A/A, A/C, or C/C genotypes (Fig. 2a). All familial BD patients had the C/C genotype, whereas HB1 and SB2 had the A/C genotype in HB1 and SB2 (Fig. 2b). *IL23R* mRNA levels (Fig. S2a) were almost identical between the A/A, A/C, and C/C genotypes (Fig. 2c). The C/C genotype was more frequent in the BD patients (red bars in Fig. 2d) than in the healthy Japanese population (black bars in Fig. 2d) (http://www.ncbi.nlm.nih.gov/SNP/snp_viewTable.cgi?pop=1411). Notably, mRNA levels of *IL12RB2* and *IL10* were unaltered (Supplementary Fig. S5). These results support a previous report that this SNV (Rs12119179) is related to BD^4^,^5^, but also indicates that it is not sufficient to cause BD symptoms.

### WES analysis of familial BD patients

**Figure 3:**
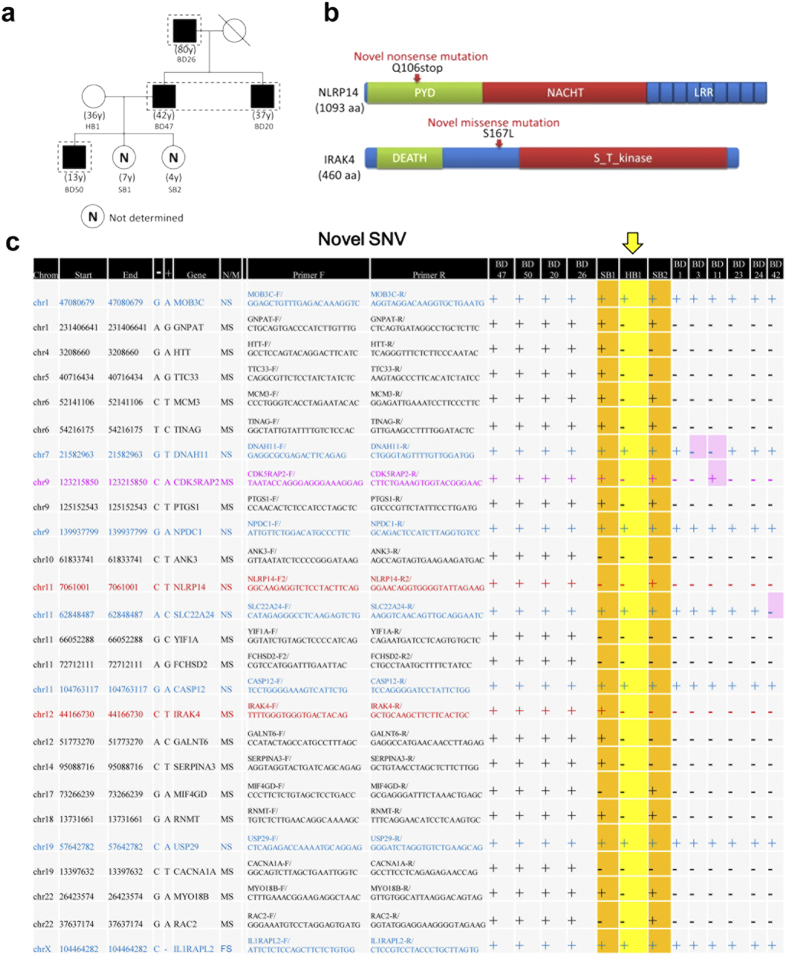
Whole-exome analysis of familial BD patients. (**a**) Pedigrees of familial BD patients (BD20, BD26, BD47, BD50, SB1, SB2, and HB1); filled symbols represent BD, empty symbols represent healthy individuals. Dashed rectangle encloses individuals that are subjected to the whole-exome analysis. Number in parentheses below each symbol indicates age of each patient. (**b**) Schematic representation of NLRP14 and IRAK4 with novel nonsense or missense mutations indicated by red arrows. (**c**) List of novel SNVs detected in one of the chromosomes of the familial BD patients (BD20, BD26, BD47, and BD50). HB1 (vertical yellow arrow) is positive (+) for the same SNVs in the genes in blue font, but negative (−) for the SNVs in the genes in black font. SNVs in pink boxes are exceptional cases. *NLRP14*, *IRAK4*, and *CDK5RAP2* are highlighted by red and pink font, respectively. Nucleotide sequences of the forward (Primer F) or reverse (Primer R) primers used for RT-PCR are shown. Chrom, chromosome number; Start or End; starting or ending coordinates of the DNA sequences on the indicated chromosome; Gene, gene symbol; NS, nonsense; MS, missense; Fs, frameshift.

Interestingly, two daughters (BD20 and BD47) and a granddaughter (BD50) of patient BD26 also suffered from BD; the husband of BD47 (HB1) was healthy, and their two sons (SB1 and SB2) were too young to diagnose for BD at the time of the study (Fig. 3a). Because cases of familial BD are rare, we performed WES analysis to determine whether these patients possess any common SNVs in protein-coding regions. On average, we generated 6.9 Gb of sequence (68,383,039 reads) per sample to a mean depth of 134×. Approximately 99.3% of reads passed quality control and were used for mapping. Ultimately, an average of 5.46 Gb of high-quality sequence (56,741,144 reads) per sample were used for SNV/indel calling, yielding a mean gross overall coverage of 106× of the target. Among these sequences, an average of 3.58 Gb of sequence mapped to the exome target. The mean capture efficiency varied across the targets: 95.5% of the target had ≥4× coverage, 90.5% had ≥10× coverage, and 82.6% had ≥20× coverage. We identified 224,391, 234,927, 233,108, and 216,409 high-quality SNVs in each of the four samples, of which 11,405 passed the filter criteria. In parallel, we predicted 15,693, 15,102, 16,280, and 16,188 small indels in these samples, of which 431 passed the filter criteria.

Because known SNVs are present in the healthy population, we searched for novel (unknown) SNVs relative to the whole human genome sequence in the data bank. In one of the alleles of the familial BD patients (BD20, BD26, BD47, and BD50), we identified 26 putative novel SNVs, including one nonsense SNV in *NLRP14* and twenty missense SNVs (Fig. 3b,c); no novel SNVs occurred in both alleles. Sanger DNA sequencing of these chromosomal regions confirmed that these heterozygous SNVs were indeed present in these BD patients, but not in HB1 (yellow vertical arrow). Some of these SNVs were either present or absent in SB1 and SB2; their physiological significance remains elusive due to the lack of clinical data at this stage.

Although *NLRP14* expression has been reported to be testis-specific^20^, NLRP14 mRNA is also detected in other tissues (http://www.genecards.org/cgi-bin/carddisp.pl?gene=NLRP14). Moreover, the family of cytosolic tripartite NLR receptors to which NLRP14 belongs plays a key role in innate immunity. The truncated NLRP14 protein encoded by the variant allele in the familial BD patients includes only a portion of the N-terminal PYD domain (Fig. 3b), which was revealed to be important in a crystallographic study^21^. Thus, NLRP14 could be involved in the pathogenesis of BD in these patients, provided that this truncated protein is not easily degraded in living cells. In addition, a missense mutation of IL-1 receptor-associated kinase 4 (*IRAK4*) (Fig. 3b) was shared by these BD patients. IRAK4 plays a pivotal role in signaling in the innate immune system^22^; therefore, this variant may also be implicated in the pathogenesis of BD. We will examine this possibility in future work.

To determine whether other BD patients also possess these SNVs, we selected five other BD patients and subjected their genomic DNA to Sanger sequencing. Most of these SNVs were not present in other BD patients, suggesting that they were specific to members of this family and not related to BD pathogenesis (Fig. 3c). One exception was *CDK5RAP2* (pink font in Fig. 3c): BD11 had an SNV (C/A) in one of her *CDK5RAP2* copies. However, the other BD patients did not possess this SNV (Supplementary Fig. S4b); thus BD11 is an exceptional case.

### *MAGEE2* SNVs in BD patients

**Figure 4:**
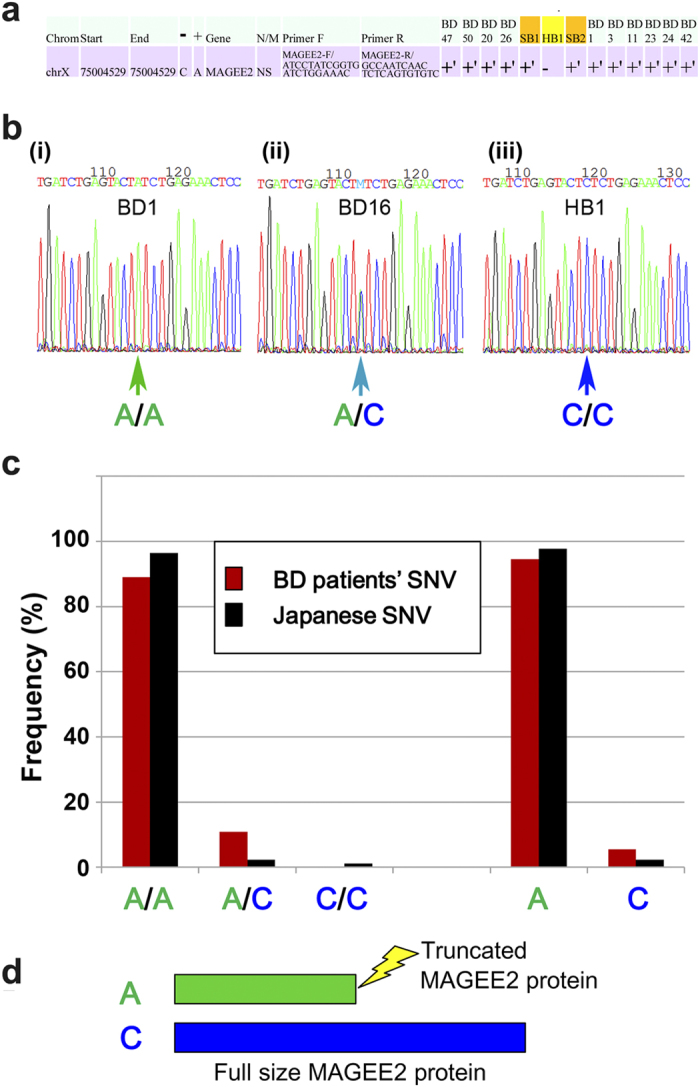
*MAGEE2* genotype and mRNA levels in BD patients. (**a**) *MAGEE2* on chromosome X contained a known SNV (A/C) in BD patients (+) but not in HB1 (−). (**b**) DNA sequence ladders around the *MAGEE2* SNV region for BD1, BD16, and HB1. Arrows indicate signal peaks for the A/A, A/C, and C/C genotype, respectively. (**c**) Percentage (%) of A/A, A/C, and C/C genotypes of *MAGEE2* in our BD patients (red bars) and the healthy Japanese population (black bars). Percentage of A and C populations calculated from A/A, A/C, and C/C genotypes are shown. (**d**) A and C genotypes generate truncated (green bar) or full-size (blue bar) MAGEE2 protein, respectively.

Another notable gene was *MAGEE2*, which was classified as a novel SNV when we obtained our WES data; we performed the SNV analysis of *MAGEE2* of because it harbored a nonsense mutation in all BD patients but not in HB1 (Fig. 4a). Although updated genomic data show that *MAGEE2* is no more a novel SNV, its variety among BD patients is notable. Indeed, Sanger DNA sequencing revealed that five other BD patients, including BD1, had the A/A genotype (Fig. 4b–i), whereas HB1 had the C/C genotype (Fig. 4b–iii). We examined the rest of the BD patients (Fig. S5B) and found that 89.1% of BD patients had the A/A genotype (Fig. 4b–i), whereas five patients (BD16, BD18, BD22, BD27, and BD38) had the A/C genotype (Supplementary Fig. S4c). The frequency of the A/C genotype in BD patients (10.9%) was higher than that in the Japanese population overall (2.3%) (http://www.ncbi.nlm.nih.gov/projects/SNP/snp_ref.cgi?rs=rs1343879). Notably, the nonsense SNV (A genotype) encodes a truncated protein, whereas the C genotype encodes the full-size protein (Supplementary Fig. S4d). These results suggest that the *MAGEE2* SNV is involved in to the pathogenesis of BD.

## Discussion

Here, we report the results of DNA microarray analyses of PBMCs from 41 Japanese BD patients. On the basis of our findings, we propose that up-regulation of *PCDH18*, *BAAT*, *CysLTR1*, and *IL23R* in PBMCs may serve as diagnostic and etiologic markers for BD (Fig. 1, S2, S3). We also performed whole-exome analysis of four familial BD patients (Fig. 2a), revealing 26 putative novel SNVs in one of the chromosomes of these patients. Notably, 19 of the novel SNVs were not present in the healthy subject HB1 (Fig. 2b,c), suggesting that these SNVs were related to BD pathogenesis in these patients. An SNV in *MAGEE2* (Fig. 4), which was present in all BD patients but not in HB1, may generate a truncated protein; likewise, the nonsense SNV in *NLRP14* encodes a truncated form of the protein (Fig. 2b) that contains only a portion of an important N-terminal domain^21^. Notably, the related protein NLRP3 regulates innate immunity as a component of the inflammasome, which is responsible for inflammatory processes through activation of caspase-1 and maturation of the inflammatory cytokines pro-interleukin-1β (pro-IL-1β) and pro-IL-18^23^. Indeed, a recombinant, non-glycosylated form of the human interleukin-1 receptor antagonist (IL-1Ra), Anakinra, is therapeutically effective in some BD patients^24^. Because IRAK4 also controls innate immunity^25^, the mutations found in both NLRP14 and IRAK4 suggests that at least a subset of BD is an auto-inflammatory syndrome caused by abnormalities in innate immunity. It remains elusive if unrelated BD patients have different mutations in the same gene; this will be examined in our future work.

Microarray analysis revealed up-regulated *IL23R* mRNA levels in BD patients (Fig. S2a). Because *IL23R* is in a susceptibility locus for BD^4^,^5^, we determined the DNA sequence around the SNV (Rs12119179) at this locus. The frequency of the C/C genotype was higher in BD patients than in healthy Japanese populations (Fig. 3). Although this result supports the relevance of this SNV to BD, it also demonstrates that the presence of this variant alone is not sufficient to cause BD symptoms. Neutrophil invasion into sites of localized inflammation (e.g., erythema nodosum, folliculitis, and genital ulcer) in the absence of infection is a notorious feature of BD. The elevation of *IL23R* mRNA in BD patients suggests that enhanced Th17 activation resulting from IL23 stimulation may be responsible for the observed neutrophil activation. Based on these considerations and the results presented in this study, we propose a new model for the pathogenesis of BD involving *IL23R*, *NLRP14*, *IRAK4* and *MAGEE2*.

## Methods

### Human subjects and ethical considerations

Forty-three BD patients were enrolled at Osaka University Hospital (BD1–41 plus the mother and daughter of BD20) between 2001 and 2009; the genders and ages of BD patients are provided in Figure S1. The study was reviewed and approved by the Research Ethics Committee of Osaka University, and written informed consent was obtained from all participants. The experimental methods involving patients were carried out in accordance with the approved guidelines. The diagnosis of BD was established according to standard criteria proposed by the Japan BD Research Committee. Serum samples were obtained from patients regardless of their symptoms and level of disease activity/inactivity.

### Target selection and sequencing

Exome sequencing was conducted on four DNA samples from patients with familial BD: patient BD47 and her aunt (BD20), grandmother (BD26), and mother (BD50). Genomic DNA was extracted from PBMC using the PAXgene Blood DNA Kit (QIAGEN), sheared into 150–200 bp fragments, and used to make a library for multiplexed paired-end sequencing (Illumina). The resultant library was hybridized to biotinylated cRNA oligonucleotide baits from the SureSelect Human All Exon 50 Mb kit (Agilent Technologies) for exome capture. Targeted sequences were purified using magnetic beads, amplified, and sequenced on an Illumina HiSeq2000 platform in paired-end 101 bp configuration. The raw sequence data were submitted to the NCBI SRA database under accession No. SRP059981 (NCBI BioProject PRJNA288379).

## Additional Information

**How to cite this article**: Okuzaki, D. *et al*. Microarray and whole-exome sequencing analysis of familial Behçet's disease patients. *Sci. Rep*. **6**, 19456; doi: 10.1038/srep19456 (2016).

## Supplementary Material

Supplementary Information
