# Supplementary material for: Microarray and whole-exome sequencing analysis of familial Behçet’s disease patients
Source: Sci Rep. 2016 Jan 20;6:19456. doi: 10.1038/srep19456 (PMC4726226; doi:10.1038/srep19456)
Supplement: Supplementary Information [file srep19456-s1.pdf]

Whole-exome sequencing analysis of familial Behçet's disease patients

Daisuke Okuzaki, Kazuyuki Yoshizaki, Toshio Tanaka, Toru Hirano, Kohshiro Fukushima, Takanori Washio, and Hiroshi Nojima

SUPPLEMENTARY INFORMATION

Supplementary Results

DNA microarray analysis

***Up-regulated genes**:* Protocadherin 18 (PCDH18) is a member of the protocadherin family, whose members function as cell surface adhesion molecules. *PCDH18* was up-regulated in all BD patients except for BD36 (**Fig. 1, Supplementary Fig. S2a, S3a**). Because this gene is expressed in memory $CD8^+$ T cells as an immediate-early gene of the memory response[30], its up-regulation in the blood cells of patients may disturb normal T-cell function via hyper-activation of CD8+ memory T cells.

Bardet–Biedl syndrome 5 (*BBS5*) is one of 18 causative genes (*BBS1–18*) for an autosomal recessive ciliopathy disorder with eye, kidney, and heart defects[31]. The BBSome (a complex of seven BBS proteins) localizes at or near the primary cilium and the centrosome, associates with the centriolar satellite protein CEP131 via BBS4, and plays a role in transporting ciliary membrane proteins[32]. BBS5, which localizes along the axonemes of rods and cones, is phosphorylated by protein kinase C to regulate the translocation of arrestin1 (Arr1) between the inner and outer segments according to the light conditions in photoreceptor cells[33]. Eye site defects observed in BD patients may be a consequence of improper translocation of Arr1 due to overexpression of *BBS5*.

Bile acid CoA:amino acid N-acyltransferase (*BAAT*) is involved in bile acid metabolism by catalyzing the hydrolysis of long- and very long-chain saturated acyl-CoAs

into the free fatty acid and coenzyme A, and also conjugates glycine to these acyl-CoAs. Defective mutations in *BAAT* are involved in familial hypercholanemia.

Cysteinyl leukotrienes (CysLT) mediate bronchoconstriction and play important roles in the development of asthma; CysLT receptor 2 (CysLTR2), a G-protein-coupled seven-transmembrane receptor for CysLTs, is expressed in multiple cell types including macrophages, vascular smooth muscle, brain, and B and T lymphocytes, in which it regulates allergic inflammation[34,35]. Notably, sequence variants of the *CYSLTR2* gene are associated with aspirin-intolerant asthma[36]. Given that augmented expression of CysLTR1 in nasal inflammatory leukocytes is also related to aspirin-intolerant asthma[37], elevated CysLTR2 expression may be involved in inflammation of various tissues in BD patients.

By contrast, no report to date has suggested any clinical relevance of several other up-regulated genes, including those encoding neuromedin U (*NMU*), guanylate cyclase 2F (*GUCY2F*), pyrophosphatase 2 (*PPA2*), and phosphoglucomutase 5 (*PGM5*). Therefore, it remains elusive why these genes were up-regulated in PBMCs of BD patients.

Down-regulated genes: mRNA levels of interferon (IFN) alpha-inducible protein 27 (*IFI27*), olfactomedin 4 (*OLFM4*), phosphatidylinositol N-acetylglucosaminyltransferase subunit C (*PIGC*), and membrane-spanning 4-domains, subfamily A, member 2 (*MS4A2*) genes were down-regulated by >-3.0–fold in both male and female BD patients relative to healthy volunteers (**Fig. 1b, Supplementary Fig. S2b, S3b**). *IFI27* encodes a mitochondrial protein that sensitizes cells to apoptotic stimuli via mitochondrial membrane destabilization, which may influence the innate immune response to IFNs[38]. Olfactomedin proteins are essential for early development and functional organization of the nervous system and hematopoiesis[39]. PIGC is an endoplasmic reticulum-associated protein involved in the first step of glycosylphosphatidylinositol lipid anchor biosynthesis[40]. MS4A2 is a subunit of the high-affinity IgE receptor (the allergic response involves the binding of allergen to

receptor-bound IgE), and SNVs in the *MS4A2* gene are potential risk factors for asthma[41]. However, scatter plot showed that average values of these genes were almost unaltered (**Supplementary Fig. S5**). Thus, we conclude that these down-regulated genes are not important.

Pathway analysis

Ficolin-1 (FCN1) functions as a recognition molecule in the complement system. We reported previously that the *FCN1* mRNA level is elevated in PBMCs from patients with systemic autoimmune diseases such as Takayasu arteritis (TA), a rare vasculitis affecting the aorta and its associated branches[42], and microscopic polyangiitis (MPA), a small-vessel vasculitis with fatal symptoms[43]. Hence, we used the Ingenuity Pathway Analysis software to investigate whether FCN1 and/or other members of the complement system are activated in BD patients. We found that the upstream factors of the classical pathway including C1r and C1s, and downstream factors including C5 and C7, are indeed up-regulated in BD patients (red circles and squares in **Supplementary Fig. S6a**), whereas other complement factors were down-regulated (green circles and diamonds in **Supplementary Fig. S6a**). In contrast to a previous study of a Chinese BD population[11], C4 protein level was not elevated in the PBMCs of our BD patients. A heat map revealed that the magnitudes of up- or down-regulation of genes encoding complement factors (**Supplementary Fig. S6b**), including *FCN1* (**Fig. S6c**), were smaller than those of the genes listed in **Fig.1**, suggesting that the complement system plays a less significant role in the pathogenesis of BD.

Analysis of other immune-related pathways revealed no notable genes, except for a modest up-regulation of defensin beta 1 (*DEFB1*) and mitogen-activated protein (MAP) kinase kinase 6 (*MAP2K6*) in many BD patients (**Supplementary Fig. S7**). *DEFB1* encodes an antimicrobial peptide that is a basic component of human innate immunity; DEFB1 plays a protective role against allergies and infection, and is up-regulated by inflammatory or

microbial stimuli[44]. Given that MAP2K6 plays a role in inflammatory diseases via phosphorylation-mediated activation of p38 MAP kinase in response to inflammatory cytokines, and MAP2K6-deficient mice exhibit reduced severity of arthritis[45], it is reasonable to speculate that its activation may influence the arthritis-related inflammatory abnormalities in some BD patients.

Supplementary Materials and Methods

Human subjects and ethical considerations

Forty-three BD patients were enrolled at Osaka University Hospital (BD1–41 plus the mother and daughter of BD20) between 2001 and 2009; the genders and ages of BD patients are provided in Figure S1. The study was reviewed and approved by the Research Ethics Committee of Osaka University, and written informed consent was obtained from all participants. The diagnosis of BD was established according to standard criteria proposed by the Japan BD Research Committee; actual study methods were carried out in accordance with the approved guidelines. Serum samples were obtained from patients regardless of their symptoms and level of disease activity/inactivity.

Target selection and sequencing

Exome sequencing was conducted on four DNA samples from patients with familial BD: patient BD47 and her aunt (BD20), grandmother (BD26), and mother (BD50). Genomic DNA was extracted from PBMC using the PAXgene Blood DNA Kit (QIAGEN), sheared into 150–200 bp fragments, and used to make a library for multiplexed paired-end sequencing (Illumina). The resultant library was hybridized to biotinylated cRNA oligonucleotide baits from the SureSelect Human All Exon 50Mb kit (Agilent Technologies) for exome capture. Targeted sequences were purified using magnetic beads, amplified, and

sequenced on an Illumina HiSeq2000 platform in paired-end 101 bp configuration. The raw sequence data were submitted to the NCBI SRA database under accession No. SRP059981 (NCBI BioProject PRJNA288379).

Mapping and SNV/indel calling

The quality of DNA reads was checked using software developed in-house. Adapter sequences and sequences of inadequate quality were removed. After quality control, reads were mapped to the reference human genome (UCSC Genome Browser, hg19) using BWA (ver. 0.5.9). Mapping results were corrected using Picard (ver. 1.49) for removal of duplicates and genome analysis toolkit (GATK; ver. 1.1-31-gdc8398e) for local alignment and quality score recalibration. SNV and insertion/deletion (indel) calls were performed on pooled reads from four samples using GATK, and filtered to coordinate with variant quality score recalibration (VQSR) passed with genotype quality score ≥ 30. Annotations of SNVs and indels were based on dbSNP135, CCDS (NCBI, November 2011 data release), RefSeq (UCSC Genome Browser, November 2011 dump), ENCODE (UCSC Genome Browser, ver. 7), and 1000Genomes (October 2011 data release). Variants were further filtered according to the following criteria: 1) genotype call existed in all four samples at the detected position; 2) predicted functions of frameshift, nonsense, read-through, missense, deletion, insertion, or insertion-deletion; 3) located outside the Segmental Duplications region (UCSC Genome Browser, November 2011 dump); and 4) located outside the Simple Tandem Repeats region (UCSC Genome Browser, November 2011 dump).

Preparation of RNA and DNA microarray analysis

RNA was prepared from PBMCs using the PAXgene Blood RNA Kit (QIAGEN). The quality of the RNA samples was examined with an RNA 6000 Nano LabChip Kit (p/n 5065-4476) on an Agilent 2100 Bioanalyzer (G2940BA; Agilent Technologies). Total RNA

(500 ng) isolated from the PBMCs of vasculitis patients was examined along with pooled RNA from healthy volunteers (Fig. S1). RNA from each patient and the pooled normal RNA were reverse-transcribed with oligo-dT primers containing the T7 RNA polymerase promoter sequence. The resulting cDNA was subjected to *in vitro* transcription with T7 RNA polymerase for Cy3 or Cy5 labeling (CyDye; Amersham Pharmacia Biotech).

Cy5-labeled cRNAs from the vasculitis patients (1,625 ng) were mixed with the same amount of reverse color Cy3-labeled cRNAs from healthy volunteers. Hybridization, rinsing, scanning, and gene analysis were conducted using Agilent Whole Human Genome Microarrays (4x44K G4112F). The ratio of Cy5/Cy3 was calculated for every probe, and the results were analyzed with the Subio Basic Plug-in (v1.6; Subio Inc.), which allows the visualization of microarray data in the form of a heat map and a line graph. Microarray data were deposited in the NCBI-GEO under the accession number GSE70403.

Pathway analysis of microarray data

Microarray data were analyzed by Ingenuity Pathways Analysis (IPA) (Ingenuity Systems,; www.ingenuity.com). Briefly, all data sets containing gene identifiers were uploaded into the application along with the corresponding expression values. Data were overlaid onto several inflammatory-related networks, which were developed using information contained in the Ingenuity Knowledge Base.

Supplementary References

30. Vazquez-Cintron, et al., Protocadherin-18 is a novel differentiation marker and an inhibitory signaling receptor for CD8+ effector memory T cells. *PLoS One* **7**, e36101; DOI: 10.1371/journal.pone.0036101 (2012).

31. Al-Hamed, et al. Functional modelling of a novel mutation in BBS5. *Cilia* **3**, 3 (2014).

32. Chamling, et al. The centriolar satellite protein AZI1 interacts with BBS4 and regulates

ciliary trafficking of the BBSome. *PLoS Genet.* **10**, e1004083; DOI: 10.1371/journal.pgen.1004083 (2014).

33. Smith, et al. Light-dependent phosphorylation of Bardet-Biedl syndrome 5 in photoreceptor cells modulates its interaction with arrestin1. *Cell Mol. Life Sci.* **70**, 4603-4616 (2013).
34. Evans, J.F. The cysteinyl leukotriene receptors. *Prostaglandins Leukot. Essent. Fatty Acids* **69**, 117-122 (2003).
35. Brink, et al. International Union of Pharmacology XXXVII. Nomenclature for leukotriene and lipoxin receptors. *Pharmacol. Rev.* **55**, 195-227 (2003).
36. Shin, et al. Genetic effect of CysLTR2 polymorphisms on its mRNA synthesis and stabilization. *BMC Med. Genet.* **10**, 106 (2009).
37. Sousa, A.R., Parikh, A., Scadding, G., Corrigan, C.J. & Lee, T.H. Leukotriene-receptor expression on nasal mucosal inflammatory cells in aspirin-sensitive rhinosinusitis. *N. Engl. J. Med.* **347**, 1493-1499 (2002).
38. Cheriyath, V., Leaman, D.W. & Borden, E.C. Emerging roles of FAM14 family members (G1P3/ISG 6-16 and ISG12/IFI27) in innate immunity and cancer. *J. Interferon Cytokine. Res.* **31**, 173-181 (2011).
39. Anholt, R.R. Olfactomedin proteins: central players in development and disease. *Front. Cell Dev. Biol.* **2**, 6 (2014).
40. Kinoshita, T. Biosynthesis and deficiencies of glycosylphosphatidylinositol. *Proc. Jpn. Acad. Ser. B Phys. Biol. Sci.* **90**, 130-143 (2014).
41. Yang, H.J., Zheng, L., Zhang, X.F., Yang, M. & Huang, X. Association of the MS4A2 gene promoter C-109T or the 7th exon E237G polymorphisms with asthma risk: a meta-analysis. *Clin. Biochem.* **47**, 605-611 (2014).
42. Okuzaki, et al. Ficolin 1 expression is elevated in the peripheral blood mononuclear cells of Takayasu's vasculitis patients. J. Mol. Biomark. Diagn. **3**, 3; DOI:

org/10.4172/2155-9929.1000125 (2012).

43. Muso, et al. Ficolin-1 is up-regulated in leukocytes and glomeruli from microscopic polyangiitis patients. *Autoimmunity* **46**, 513-524 (2013).
44. Prado-Montes de Oca, E. Human beta-defensin 1: a restless warrior against allergies, infections and cancer. *Int. J. Biochem. Cell Biol.* **42**, 800-804 (2010).
45. Hammaker, et al. Decreased collagen-induced arthritis severity and adaptive immunity in MKK-6-deficient mice. *Arthritis. Rheum.* **64**, 678-87 (2012).

List of Abbreviations

Bardet–Biedl syndrome 5 (BBS5); Behçet's disease (BD); bile acid CoA:amino acid N-acyltransferase (BAAT); copy-number variation (CNV); cysteinyl leukotrienes (CysLT); CysLT receptor 1 (CysLTR1); CysLT receptor 2 (CysLTR2); defensin beta 1 (DEFB1); ficolin-1 (FCN1); genome-wide association study (GWAS); GTPases of immunity-associated protein (GIMAP); guanylate cyclase 2F (GUCY2F); interferon (IFN) alpha-inducible protein 27 (IFI27); interleukin (IL) 23 receptor (IL23R); IL-1 receptor-associated kinase 4 (IRAK4); membrane-spanning 4-domains, subfamily A, member 2 (MS4A2); microscopic polyangiitis (MPA); mitogen-activated protein (MAP); MAP kinase kinase 6 (MAP2K6); melanoma antigen-encoding gene E2 (MAGEE2); nucleotide-binding oligomerization domain (NOD)-like receptor family pyrin domain-containing 14 (NRP14); neuromedin U (NMU); olfactomedin 4 (OLFM4); peripheral blood mononuclear cell (PBMC); phosphatidylinositol N-acetylglucosaminyltransferase subunit C (PIGC); phosphoglucomutase 5 (PGM5); protocadherin 18 (PCDH18); pyrophosphatase 2 (PPA2); single-nucleotide polymorphism (SNP); single-nucleotide variation (SNV); Takayasu arteritis (TA); whole-exome sequencing (WES).

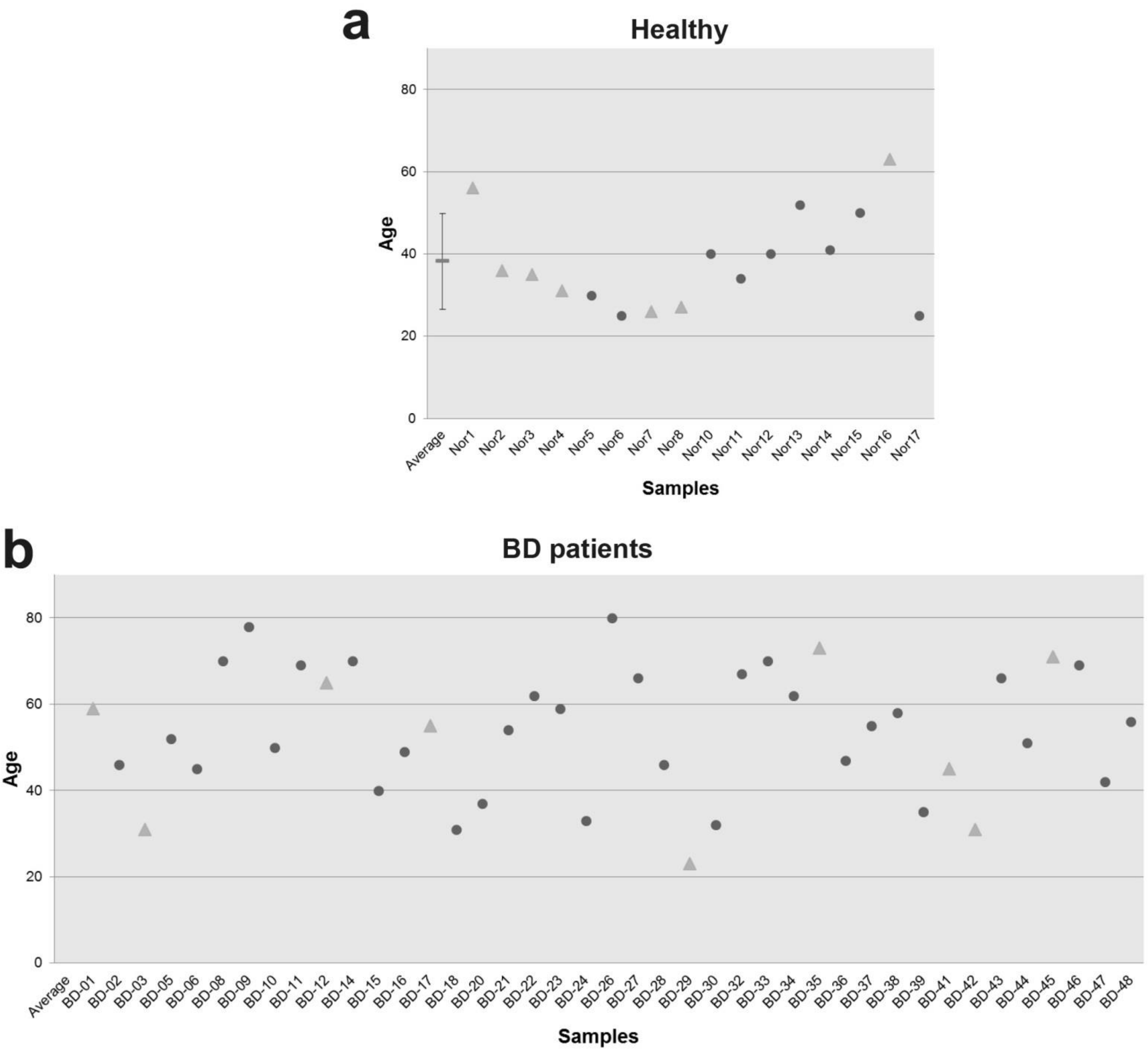

Figure S1. Gender and age distributions of healthy volunteers (a) and BD patients (b). Females and males are indicated by circles and triangles, respectively.

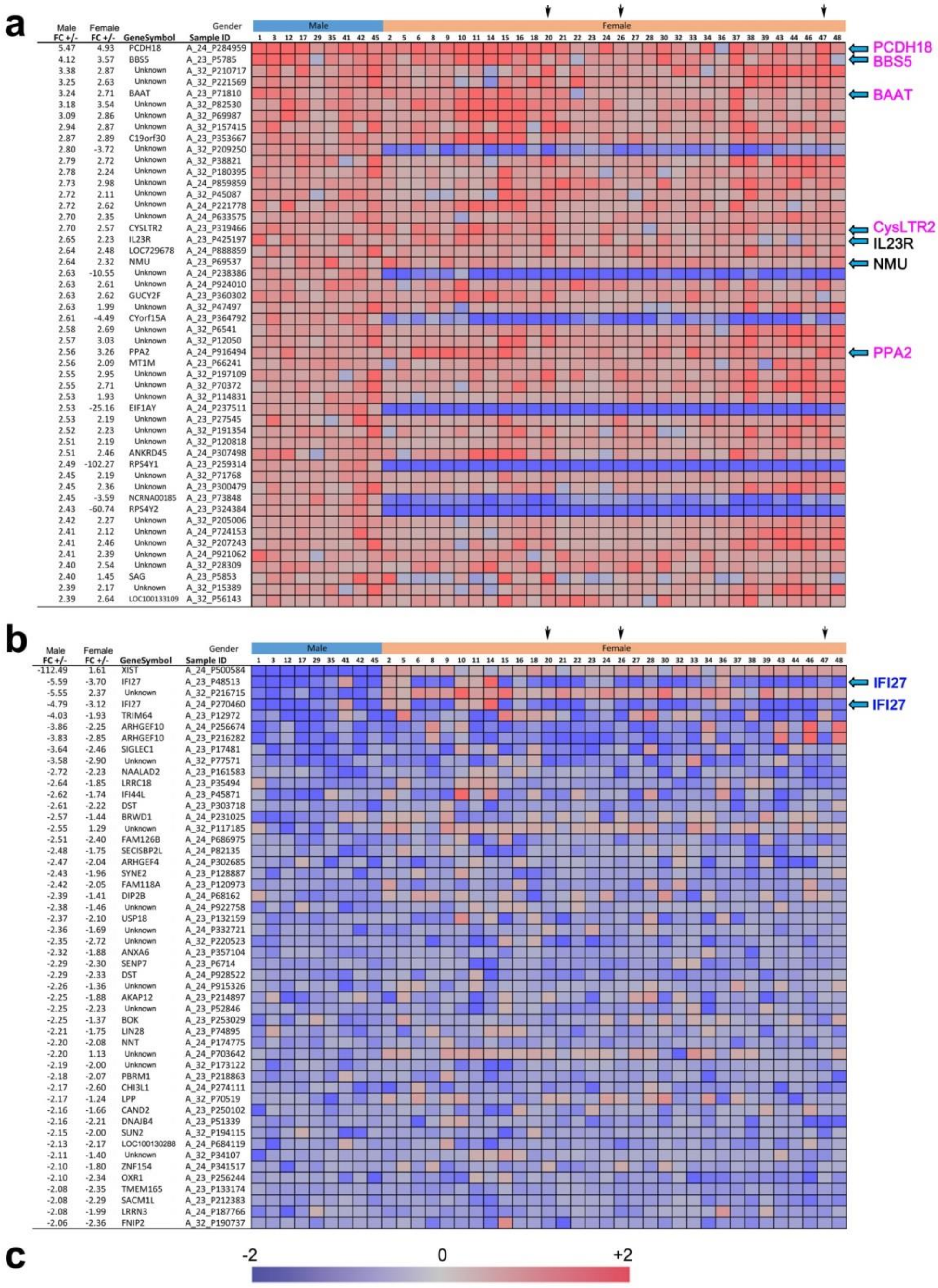

Figure S2. Expression profiles of genes whose mRNA levels were commonly up- or down-regulated in the PBMCs of 41 BD patients relative to those of healthy volunteers. Agilent's whole human genome DNA microarray (Hu44K) was used for this analysis. **(a)** List of top 50 genes up-regulated in most BD patients is shown in decreasing order of fold-change values for male patients. **(b)** List of bottom 50 genes down-regulated in most BD patients is shown in increasing order of fold-change values for male patients. "Unknown" indicates uncharacterized genes. Agilent's sample ID is presented for identification of probes used for the microarray analysis. Mosaic tile representation of each gene is also shown, with intensity gradients indicating the mean value of the expression level (log_2 ratio): blue (down-regulation) and crimson (up-regulation) relative to the average value in healthy volunteers (gray). Names of notable genes are highlighted in larger font with blue arrows; genes also appearing in Figure S2 are shown in pink font. Vertical arrows indicate familial BD patients (see Fig. 2A). **(c)** Bar represents the standard intensity gradient.

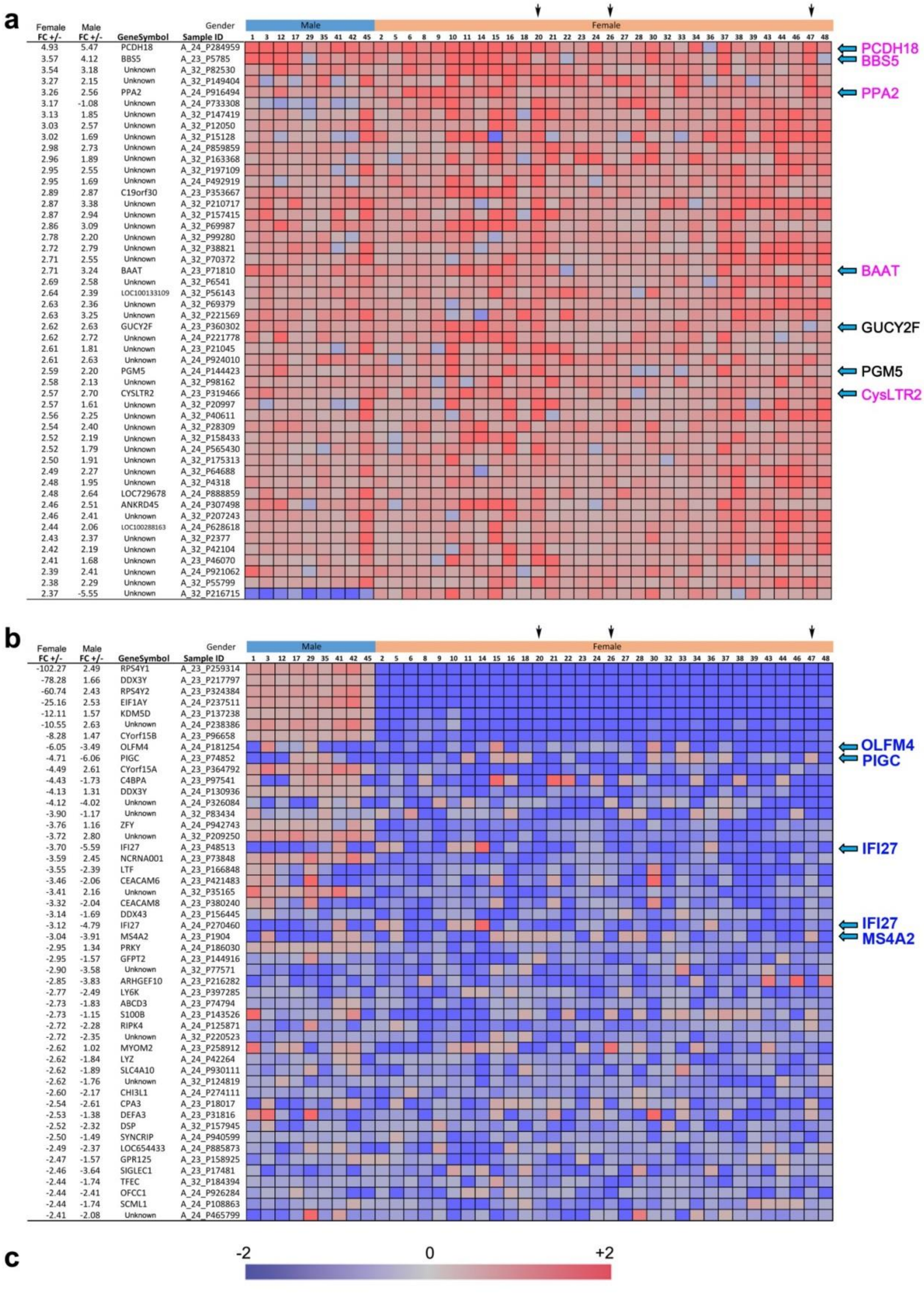

Figure S3. Expression profiling of microarray data for 41 BD patients. (a) List of top 50 genes up-regulated in most BD patients is shown in decreasing order of fold-change values for female patients. **(b)** List of bottom 50 genes down-regulated in most BD patients is shown in increasing order of fold-change values for female patients. "Unknown" indicates uncharacterized genes. Agilent's sample ID is presented for identification of probes used for the microarray analysis. Mosaic tile representation of each gene is also shown, with intensity gradients indicating the mean value of the expression level (log_2 ratio): blue (down-regulation) and crimson (up-regulation) relative to the average value in healthy volunteers (gray). Names of notable genes are highlighted in larger font with blue arrows; genes also appearing in Figure S2 are shown in pink font. Vertical arrows indicate familial BD patients (see Fig. 2a). **(c)** Bar represents the standard intensity gradient.

a

IL23R

Patient Number	Array data	Geno-type
BD1	5.61	AA
BD2	3.00	AC
BD3	1.20	AA
BD5	1.50	CC
BD6	3.69	AC
BD8	4.25	AC
BD9	2.21	CC
BD10	4.78	AC
BD11	3.91	AC
BD12	4.42	AA
BD14	2.88	CC
BD15	2.59	AA
BD16	2.79	AC
BD17	3.23	AC
BD18	2.04	AA
BD20	5.69	CC
BD21	1.65	AC
BD22	1.76	CC
BD23	1.53	AC
BD24	4.63	AC
BD26	1.01	CC
BD27	2.58	CC
BD28	1.29	CC
BD29	1.50	AA
BD30	4.02	CC
BD32	2.07	CC
BD33	1.23	CC
BD34	2.54	CC
BD35	1.94	AA
BD36	3.26	AA
BD37	1.97	CC
BD38	2.26	AC
BD39	-1.07	CC
BD41	5.54	CC
BD42	2.24	AC
BD43	1.57	CC
BD44	-1.97	CC
BD45	1.84	Nd
BD46	2.18	Nd
BD47	1.92	CC
BD48	2.36	CC
BD50	nd	CC
SB1	nd	CC
SB2	nd	AC
HB1	nd	AC

b

CDK5Rap2

Patient Number	Geno-type
BD1	CC
BD2	CC
BD3	CC
BD5	CC
BD6	CC
BD8	CC
BD9	CC
BD10	CC
BD11	AA
BD12	CC
BD14	CC
BD15	CC
BD16	CC
BD17	CC
BD18	CC
BD20	AA
BD21	CC
BD22	CC
BD23	CC
BD24	CC
BD26	AA
BD27	CC
BD28	CC
BD29	CC
BD30	CC
BD32	CC
BD33	CC
BD34	CC
BD35	CC
BD36	CC
BD37	CC
BD38	CC
BD39	CC
BD41	CC
BD42	CC
BD43	CC
BD44	CC
BD45	Nd
BD46	Nd
BD47	AA
BD48	Nd
BD50	AA
SB1	AA
SB2	AA
HB1	CC

c

MAGEE2

Patient Number	Geno-type
BD1	AA
BD2	AA
BD3	AA
BD5	AA
BD6	AA
BD8	AA
BD9	AA
BD10	AA
BD11	AA
BD12	AA
BD14	AA
BD15	AA
BD16	AC
BD17	AA
BD18	CC
BD20	AA
BD21	AA
BD22	AC
BD23	AA
BD24	AA
BD26	AA
BD27	AC
BD28	AA
BD29	AA
BD30	AA
BD32	AA
BD33	AA
BD34	AA
BD35	AA
BD36	AA
BD37	AA
BD38	AC
BD39	AA
BD41	AA
BD42	AA
BD43	AA
BD44	AA
BD45	Nd
BD46	Nd
BD47	AA
BD48	Nd
BD50	AA
SB1	AA
SB2	AA
HB1	AA

Figure S4. Genotypes of SNVs for *IL23R, CDK5Rap2* and *MAGEE2* genes for BD patients. Familial BD patients are highlighted by red font. Nd, not determined due to lack of DNA samples. Array data (fold change) are shown for IL23R gene.

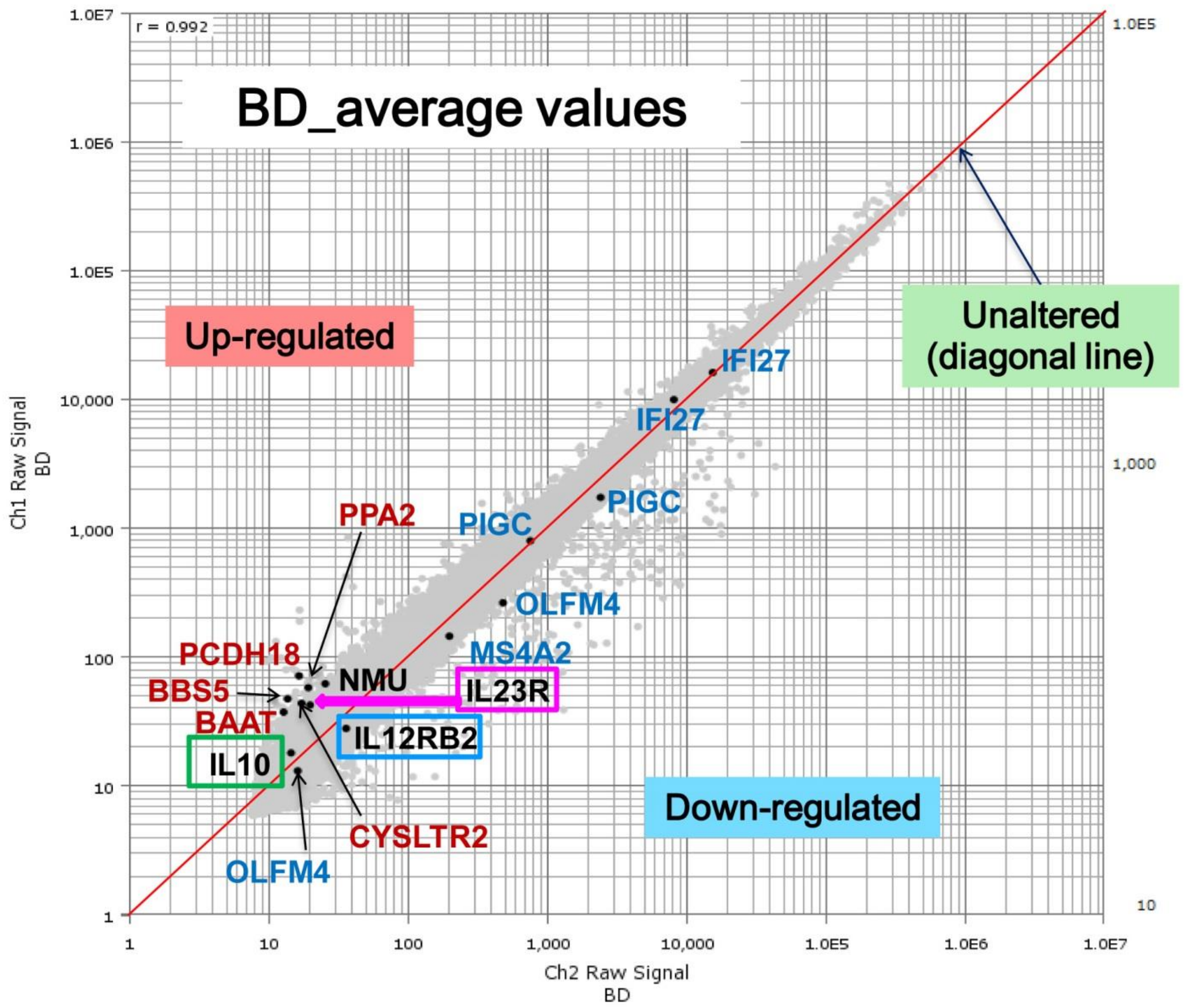

Figure S5. Scatter plots of highlighted genes in Fig. 1, S2 and S3. The y-axis shows the log value of hybridization signal intensity obtained from the microarray data for BD patients (average values). The x-axis shows the log value of signal intensity obtained from samples of healthy volunteers. Dots for *IL12RB2* and *IL10* are also shown to compare with *IL23R* dot.

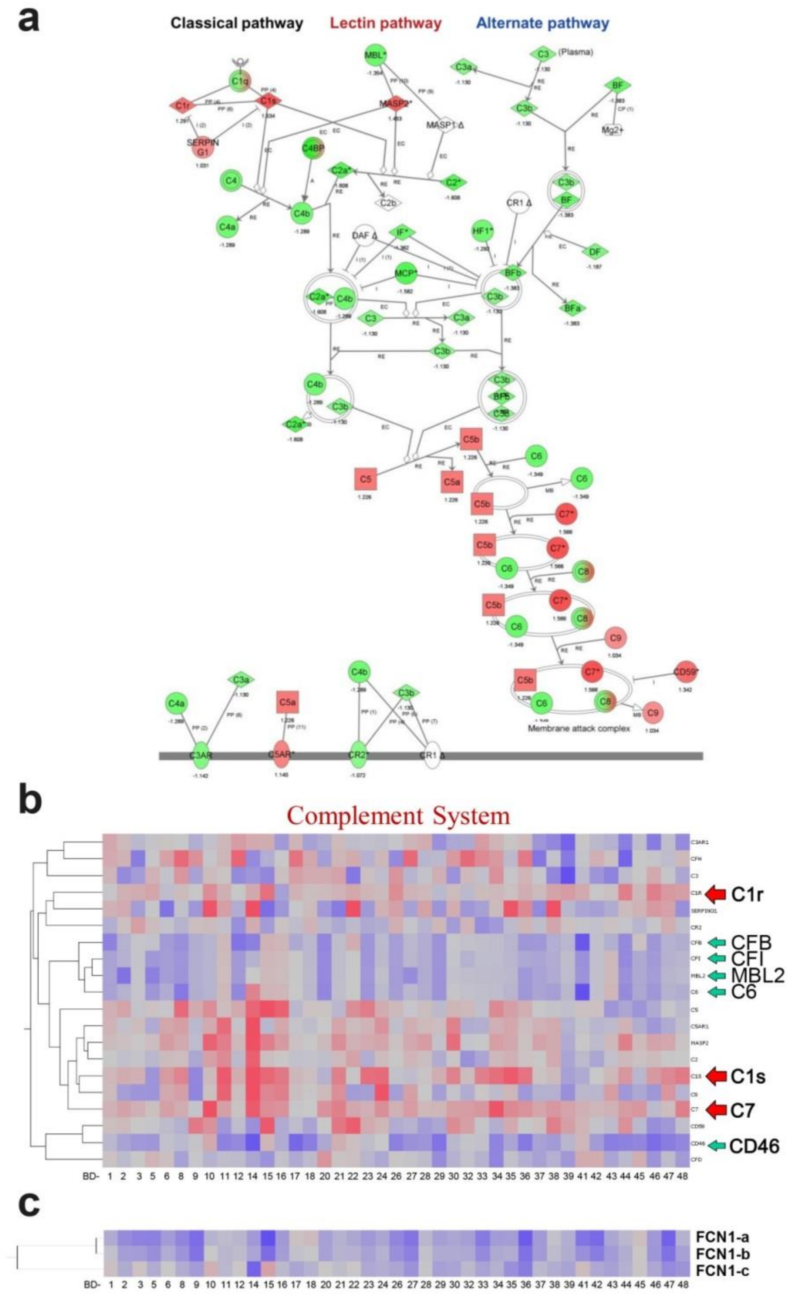

Figure S6. Expression profiling of microarray data for complement system genes expressed in PBMCs of 41 BD patients. (a) Ingenuity Pathway Analysis of microarray data for complement system genes identified *C5* and *C7* as the most conspicuously up-regulated genes in all 41 BD patients. The network shows a graphical representation of the molecular relationships between molecules, which are represented as nodes. Edges (lines) represent the biological relationship between two nodes; all edges are supported by at least one reference from the literature, a textbook, or canonical pathway information stored in the Ingenuity Knowledge Base. Node shapes represent the functional classes of the gene products, and node color intensity indicates the degree of up- (red) or down- (green) regulation. Edges are labeled in various ways that describe the nature of the relationships between nodes. **(b)** Mosaic tile representation of genes involved in the complement system. Notable genes are highlighted in large font and by red (up-regulated) or green (down-regulated) arrows. **(c)** Mosaic tile representation of three independent *FCN1* probes. Tile colors indicate the mean relative transcript levels in PBMCs from BD patients and normal controls. Blue corresponds to a $\log_2$ ratio of -5 (down-regulation), red corresponds to a $\log_2$ ratio of 5 (up-regulation), and intermediate values are represented by shades of red (pink) or blue. Gray indicates no change.

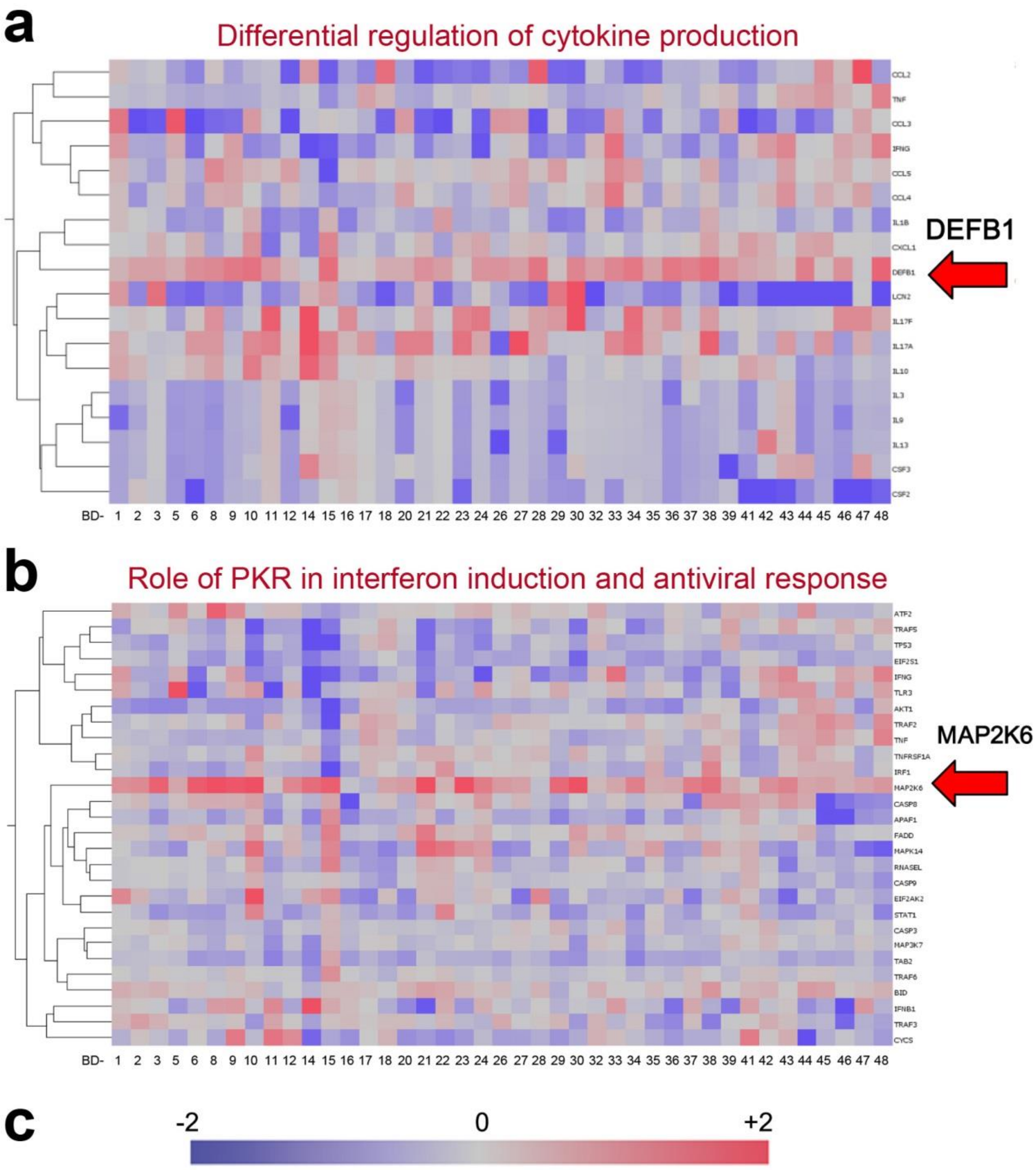

Figure S7. Expression profiling of *DEFB1* and *MAP2K6* genes in PBMCs of 41 BD patients. (a) Mosaic tile representation of genes involved in the "differential regulation of cytokine production" pathway. *DEFB1*, which was up-regulated in many BD patients, is highlighted by a red arrow. **(b)** Mosaic tile representation of genes involved in the "role of protein kinase receptor (PKR) in interferon induction and antiviral response" pathway. *MAP2K6*, which is up-regulated in many BD patients, is highlighted by a red arrow. **(c)** Intensity gradients indicate the mean value of the expression level (log_2 ratio): down-regulation (blue) and up-regulation (crimson) are shown relative to the average value of healthy volunteers (gray).
